# Unusual presentation of a hepatocellular carcinoma as a potential late side effect of radiotherapy in a patient treated for Wilms tumor in childhood

**DOI:** 10.1186/s12957-018-1346-1

**Published:** 2018-03-07

**Authors:** Deborah Repullo, Marie Diaz, Stéphane Holbrechts, Maria Gomez-Galdón, Dirk Van Gestel, Ali Bohlok, Gabriel Liberale, Vincent Donckier

**Affiliations:** 1 Service de Chirurgie, Institut Jules Bordet, Université Libre de Bruxelles, 121 Boulevard de Waterloo, 1000 Brussels, Belgium; 2 Service d’Oncologie, CHU Ambroise Paré, 2 Boulevard Kennedy, 7000 Mons, Belgium; 3 Service d’Anatomie Pathologique, Institut Jules Bordet, Université Libre de Bruxelles, 121 Boulevard de Waterloo, 1000 Brussels, Belgium; 4 Service de Radiothérapie, Institut Jules Bordet, Université Libre de Bruxelles, 121 Boulevard de Waterloo, 1000 Brussels, Belgium

**Keywords:** Radiotherapy-induced, Hepatocellular carcinoma, Wilms tumor

## Abstract

**Background:**

The development of a second primary tumor is a potential late side effect of radiotherapy. Particularly, an increased risk of secondary cancers, mostly of digestive or breast origin, has been observed in patients treated with high-dose radiotherapy for Wilms tumor (WT) in childhood. However, hepatocellular carcinoma (HCC) has been very rarely described as a potentially radiotherapy-induced tumor. We describe the case of a patient with an aggressive HCC 50 years after the treatment of a WT.

**Case presentation:**

A 49-year old man, treated at the age of 6 weeks for a right WT by a right nephrectomy and adjuvant radiotherapy, presented with a right abdominal mass. Imaging demonstrated a 100-mm tumor invading the inferior segment of the right liver, the right colon and the right psoas muscle. The patient had no previous history of liver disease, nor of alcohol consumption, and hepatitis serologies were negatives. Biopsy demonstrated a poorly differentiated tumor of unknown origin. A panel of tumor markers was negative. Explorative surgery has been performed allowing en bloc R0 tumor resection, including resection of segments VI and VII of the liver, right hemicolectomy and resection of the anterior sheet of the right psoas muscle. Pathological examination revealed a poorly differentiated HCC. No signs of cirrhosis or chronic liver disease were observed in the non-tumor liver. Twenty weeks after surgery, the patient developed a multifocal tumor recurrence that was treated with intra-arterial ^90^Yttrium radioembolization.

**Conclusion:**

In this case, the absence of risk factors for HCC, such as cirrhosis, viral hepatitis and chronic liver disease, highly suggests the development of HCC to be related to previous high-dose radiation therapy given for a right WT to a field involving the inferior part of the liver. This observation shows radiotherapy to/near the liver, particularly in childhood, to be a potential risk factor for HCC, stressing the need for a long-term specific follow-up in patients irradiated in childhood.

## Background

Radiotherapy is a well-known oncogenic factor, leading to an increased risk of secondary neoplasms in patients who received irradiation for the treatment of a cancer, especially when this treatment is given at young age [1]. Such risk of secondary neoplasms has been reported in several conditions in organs included within the field of irradiation and proportional to the administered doses [1]. Particularly, an increased risk of secondary cancers has been reported in adult patients treated with radiotherapy for a Wilms tumor (WT) in childhood [2–4]. These tumors arise 40 to 50 years after radiotherapy and are predominantly of digestive and breast origins [2]. The risk factors for hepatocellular carcinoma (HCC) are well-established and are mostly related to hepatitis viruses, chronic liver diseases and cirrhosis. The potential role of liver irradiation to promote HCC development has been poorly documented and only rare cases of HCC after previous radiotherapy involving the liver have been described [5–7]. We report on the case of a patient without previous liver disease who developed an aggressive form of HCC 50 years after surgery and adjuvant radiotherapy for a right WT, illustrating the potential causal relationship between radiation to the liver and development of primary liver cancer.

## Case presentation

**Fig. 1:**
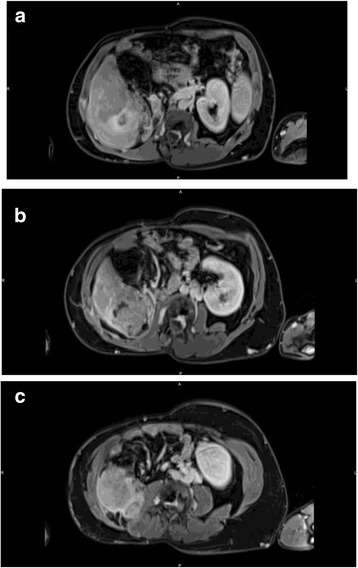
Venous phase CT scanner showing a right flank tumor invading segment VI of the liver (**a**), right kidney fossa, anterior sheet of right psoas muscle and right colon (**b** and **c**)

A 49-year old man presented with a right abdominal pain, peaks of fever and significant weight loss. His medical history was marked by a right WT at the age of 6 weeks, treated by right nephrectomy followed by adjuvant regional radiotherapy. The precise details on the administered radiotherapy could not be found but most of the schemes applied at this time consisted of relatively large fields to intermediate doses. The complete work-up included an abdominal CT scan and a magnetic resonance imaging showing a 100-mm mass in the right flank, invading the hepatic flexure of the right colon, the segment VI of the liver and the right psoas muscle (Fig. 1a–c). The tumor appeared hypervascularized on the arterial phase, displaying a wash-out on the portal phase. Colonoscopy showed a tumor invading the right colon lumen, described as from extrinsic origin. Multiple biopsies were performed during colonoscopy, showing a poorly differentiated tumor of unknown origin. Tumor markers, including CEA, CA19.9 and alpha-fetoprotein, were negative. Of note, the patients had no history of liver disease, nor of alcohol consumption, serology for hepatitis A, B and C was negative and liver imaging did not show any sign of cirrhosis. Moreover, previous abdominal CT scan performed 5 years before in the context of abdominal occlusion did not reveal any liver lesion. On this basis and in the absence of any other secondary location, an exploratory laparotomy has been planned.

**Fig. 2:**
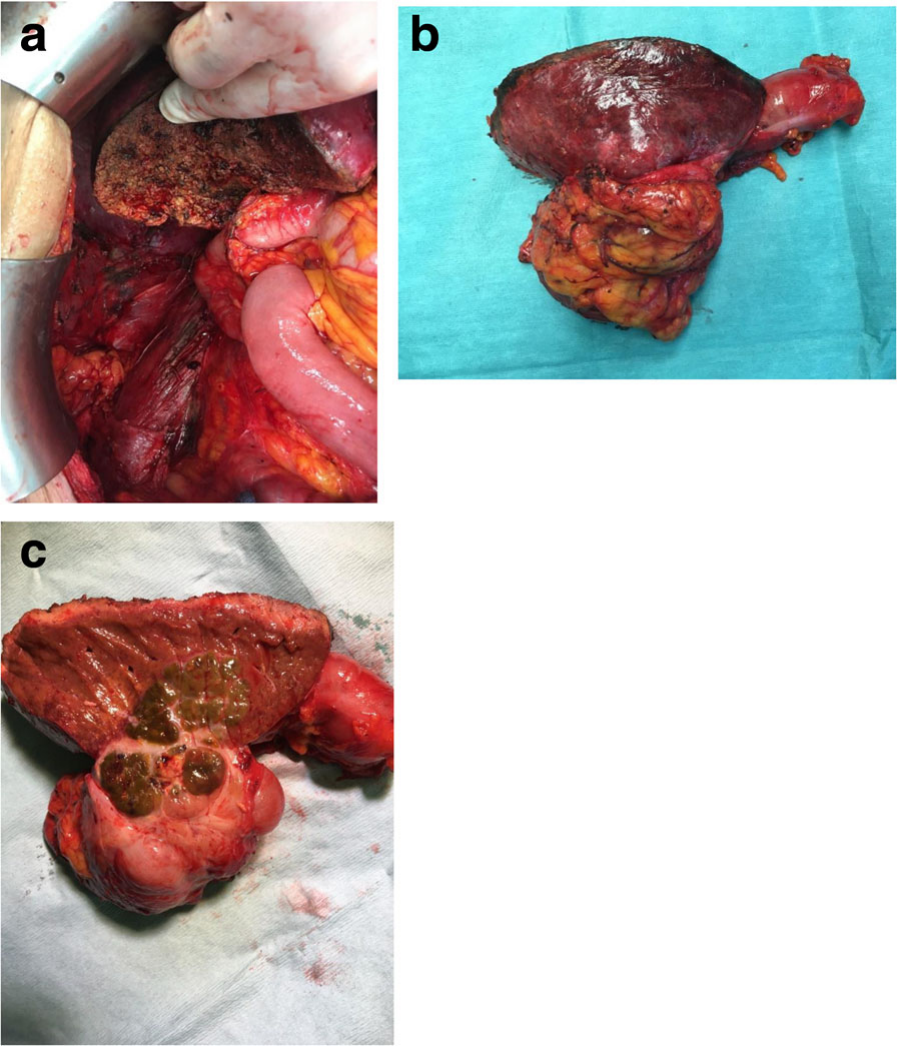
**a** Operative field after en bloc resection of segments VI and XII of the liver, right hemicolectomy and resection of anterior sheet of right psoas muscle. **b** Macroscopic view of operative specimen. **c** Section of the operative specimen showing the liver tumor with extrinsic invasion of the hepatic flexure of the right colon

**Fig. 3:**
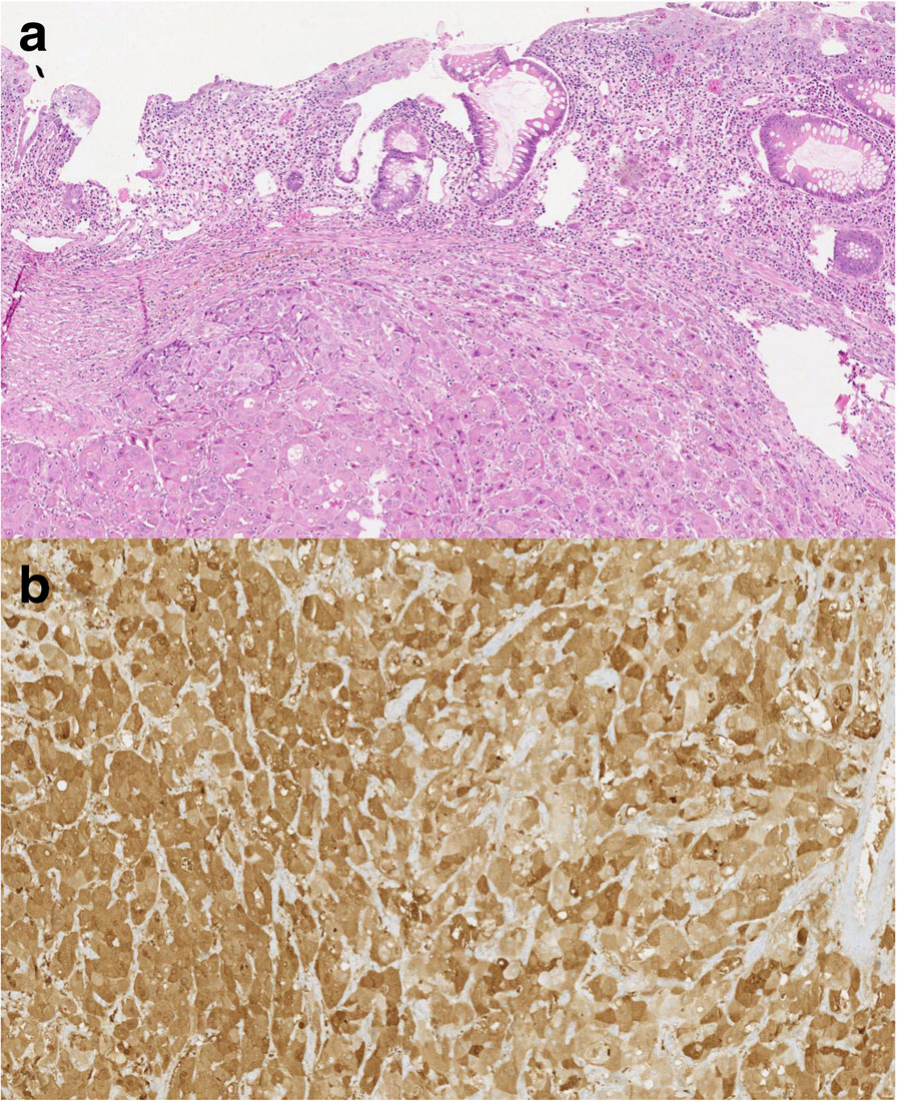
**a** Pathological examination showing a typical poorly differentiated hepatocellular carcinoma. **b** Arginine-positive staining confirming the diagnosis of hepatocellular carcinoma

At surgery, an invasive tumor at the right flank was found, involving the segment VI of the liver and the hepatic colonic flexure. No plane of dissection could be observed between the tumor and the psoas muscle. Accordingly, en bloc resection including right hemicolectomy, resection of liver segments V and VI and resection of the anterior sheet of psoas muscle has been performed (Fig. 2a–c). Postoperative course was uneventful and patient was discharged on postoperative day 12. Pathologic examination demonstrated a poorly differentiated HCC (Fig. 3a) and a margin-free (R0) resection. Arginine-positive staining confirmed the diagnosis of HCC (Fig. 3b). Of note, the liver at distance of the tumor appeared of normal structure, without any signs of cirrhosis or other liver damage. Twenty weeks after surgery, the control CT scanner showed a multifocal recurrence. At this time, the patient was proposed for whole-liver ^90^Yttrium intra-arterial radioembolization.

## Discussion

Radiotherapy is a known risk factor for second primary cancers in the field of irradiation. Since this risk increases when treatment has been given in childhood and since it is proportional to the dose of irradiation, this complication has been particularly observed in patients treated for WT in childhood as treatment in the past consisted in the combination of surgery and relatively high-dose adjuvant radiotherapy [2]. HCC has rarely been evocated as induced by previous radiotherapy, and only few cases of potentially radiotherapy-induced HCC have been described, after lombo-aortic irradiation for seminoma [5] or after extended field irradiation for Hodgkin’s lymphoma [6]. Similarly to the present case, Kovalic described four cases of HCC among a total population of more than 14,000 patients treated for WT in childhood [7]. All four patients had right-sided WT and were treated with adjuvant right upper quadrant abdominal radiotherapy. They developed HCC 15 to 20 years after treatment for WT and they all died shortly after HCC diagnosis.

In the present case, several factors suggest previous locoregional radiotherapy to be the predominant oncogenic factor in the development of HCC. First, the field of irradiation used as adjuvant therapy for right WT is likely to have included the inferior segments of the right liver (segments V and VI) which appeared as the tumor site. Secondly, besides previous regional irradiation, the patient had no other risk factors for HCC. Thirdly, the tumor occurred 50 years after irradiation, corresponding to the peak of incidence as described for radiotherapy-induced tumors [2]. Finally, the clinical presentation in this case was unusual for HCC, suggesting that its development could correspond to a specific pathogenesis [7]. Indeed, the tumor appears highly aggressive, invading adjacent structures, corresponding to a poorly differentiated HCC and leading to rapid multifocal recurrence despite R0 resection.

## Conclusion

This observation underlines the increased risk for second primary cancers in patients who received high-dose radiotherapy for WT in childhood and suggests that HCC could be a rare radiation-induced tumor when the liver had been included in the field of irradiation. This case underscores the need for a prolonged surveillance of such patients, with a particular attention to the liver when included in the field of radiotherapy.

## Abbreviations

HCC: Hepatocellular carcinoma
WT: Wilms tumor
